# Perilla Leaf Extract Attenuates Asthma Airway Inflammation by Blocking the Syk Pathway

**DOI:** 10.1155/2021/6611219

**Published:** 2021-05-10

**Authors:** Hui Yang, Wei Sun, Yan-nan Fan, Shu-yi Li, Ji-qiao Yuan, Zi-qian Zhang, Xu-yu Li, Ming-bao Lin, Qi Hou

**Affiliations:** ^1^State Key Laboratory of Bioactive Substances and Functions of Natural Medicines, Institute of Materia Medica, Chinese Academy of Medical Sciences & Peking Union Medical College, Beijing, China; ^2^Beijing Friendship Hospital, Capital Medical University, Beijing, China

## Abstract

*Perilla frutescens* (L.) Britton is a classic herbal plant used widely against asthma in China. But its mechanism of beneficial effect remains undermined. In the study, the antiallergic asthma effects of Perilla leaf extract (PLE) were investigated, and the underlying mechanism was also explored. Results showed that PLE treatment significantly attenuated airway inflammation in OVA-induced asthma mice, by ameliorating lung pathological changes, inhibiting recruitment of inflammatory cells in lung tissues and bronchoalveolar lavage fluid (BALF), decreasing the production of inflammatory cytokines in the BALF, and reducing the level of immunoglobulin in serum. PLE treatment suppressed inflammatory response in antigen-induced rat basophilic leukemia 2H3 (RBL-2H3) cells as well as in OVA-induced human peripheral blood mononuclear cells (PBMCs). Furthermore, PLE markedly inhibited the expression and phosphorylation of Syk, NF-*κ*B, PKC, and cPLA_2_ both *in vivo* and *in vitro*. By cotreating with inhibitors (BAY61-3606, Rottlerin, BAY11-7082, and arachidonyl trifluoromethyl ketone) *in vitro*, results revealed that PLE's antiallergic inflammatory effects were associated with the inhibition of Syk and its downstream signals NF-*κ*B, PKC, and cPLA_2_. Collectively, the present results suggested that PLE could attenuate allergic inflammation, and its mechanism might be partly mediated through inhibiting the Syk pathway.

## 1. Introduction

Asthma is one of the most common respiratory diseases characterized by varying degrees of chronic airway inflammation [1]. With increasing prevalence, it causes 250,000 deaths annually and will affect approximately 400 million individuals globally by 2025 [1, 2]. The most effective anti-inflammatory drugs used in asthma are inhaled corticosteroids (ICS) to suppress airway inflammation [3]. Unfortunately, even with the maximal medical therapy, over half of asthmatic patients do not achieve adequate medical control [4, 5]. The intake of ICS is also associated with numerous adverse effects, including impaired growth in children, suppressed hypothalamic-pituitary axis, and increased risk of infections [6]. Thus, there is an urgent need to develop novel anti-inflammatory drugs for asthma treatment.

Traditional Chinese medicine has been widely used in treating asthma in clinic for many years due to the ability to inhibit allergic hyperreactivity and regulate immune balance [7]. *Perilla frutescens* (L.) Britton, an annual classic herb that belongs to the *Lamiaceae* mint family [8], exerts great anti-inflammation and immune-regulation effects [9]. However, the therapeutic effects and potential mechanism of Perilla leaves on asthma have not yet been fully elucidated.

Spleen tyrosine kinase (Syk) is a 72 kD cytoplasmic nonreceptor tyrosine kinase, which plays key roles in allergic asthma inflammatory responses [10] through promoting IgE activation, degranulation mediator release, eicosanoid production, and cytokine synthesis [11, 12]. Syk activation occurs primarily through SH2 domains binding to Fc*ε*RI-ITAMs (immunoreceptor tyrosine-based activation motifs) [13] and then phosphorylating its own activation loop in tyrosine 525/526 to fully activate itself and transduce Fc*ε*RI signaling in mast cells and basophils [14]. As an upstream signaling molecule, activated Syk regulates multiple signaling molecules and amplifies inflammatory signals [15], including nuclear factor-*κ*B (NF-*κ*B), protein kinase C (PKC), and cytosolic phospholipase A_2_ (cPLA_2_) [16]. Recently, Syk inhibitors have been considered to constitute a new anti-inflammatory treatment strategy for asthma [14]. Therapies by inhibiting Syk may be more effective than treatments that inhibit a single downstream event.

In the present study, by using OVA-induced asthma mouse model, OVA-induced human PBMC inflammation model, and DNP-IgE/BSA-induced RBL-2H3 cell model, the anti-inflammatory effects of PLE on asthma were investigated. In addition, its mechanism of targeting Syk and downstream signals was explored.

## 2. Materials and Methods

### 2.1. Materials and Reagents

OVA, DNP-IgE, PIPES, 4-nitrophenyl-N-acetyl-*β*-D-glucosamide, BAY61-3606, Rottlerin, and BAY11-7082 were purchased from Sigma-Aldrich (MO, USA). Arachidonyl trifluoromethyl ketone (ATK) was from Cayman Chemical (MI, USA). Dexamethasone sodium phosphate (Dex) was purchased from Rongsheng Pharmaceutical Co., Ltd. (Jiaozuo, China). Aluminum hydroxide gel was from Meihua chemical industry limited company (Shanghai, China). All enzyme-linked immunosorbent assay (ELISA) kits were from BioLegend (CA, USA). ABC kit was obtained from Zhongshan Golden Bridge Biotechnology Co, Ltd. (Beijing, China). Lymphocyte Separation Medium was from Haoyang Biological Manufacture Co., Ltd. (Tianjin, China). RPMI-1640 medium, DMEM medium, and FBS were from Gibco (NY, USA). DNP-BSA was from Biosearch (CA, USA). Toluidine blue was from Amresco (Ohio, USA). Antibodies against Syk, p-Syk (Y525/526), PKC, p-PKC, NF-*κ*B p65, p-NF-*κ*B p65 (Ser536), cPLA_2_, p-cPLA_2_ (Ser505), and *β*-actin were obtained from Cell Signaling Technology (MA, USA). The hemocytometer was from Mindray BC-5000 vet (Shenzhen, China). The inverted research microscope was from Nikon Eclipse Ti2 (Japan). PLE was prepared as described in previous study, and the major compounds in PLE were roseoside, vicenin-2, and rosmarinic acid [17].

### 2.2. Animals

Male *Balb/c* wild-type mice (18-20 g, 6-8 weeks old, Beijing Vital River Laboratory Animal Technology Co., Ltd., Beijing, China) were raised under controlled temperature (24 ± 2°C), humidity (60 ± 5%), and photoperiod (12 h light/dark cycle). Standard laboratory chow diet and water were provided *ad libitum*.

### 2.3. Ethics Declarations and Approval for Animal Experiments

All the animal experiments were carried out according to the Standard Operating Procedure for Animal Experimental Center, Institute of Materia Medica (IMM), Chinese Academy of Medical Sciences & Peking Union Medical College (CAMS & PUMC), approved by the Animal Care &Welfare Committee of IMM, CAMS & PUMC, and conformed to internationally accepted ethical standards.

### 2.4. OVA-Induced Allergic Airway Inflammation and Treatment

The OVA-induced asthma mouse model was established according to a previously described method [17]. Briefly, animals were divided randomly into 6 groups: control (Con) group, model (Mod) group, PLE 100 mg/kg group, PLE 200 mg/kg group, PLE 400 mg/kg group, and Dex 0.5 mg/kg [18] group. On days 1, 7, and 14, mice in the model group and experimental groups were sensitized with intraperitoneal injection of 30 *μ*g OVA adsorbed in 1 mg aluminum hydroxide gel [19]. Mice were intragastrically administrated with vehicle or varying treatments on days 15-28 and challenged with intratracheal instillation of 80 *μ*g OVA on days 26, 27, and 28. Animals were euthanized on day 29.

### 2.5. Measurement of Cell Counts in Bronchoalveolar Lavage Fluid (BALF)

Mice were sacrificed by cervical dislocation 24 h after the last OVA challenge, and BALF was obtained by intratracheal instillation with 700 *μ*l PBS triply. The supernatant was collected after centrifugation at 4°C for the determination of TNF-*α*, IL-4, IL-6, and IFN-*γ* by ELISA kits. The cell pellets were resuspended in 500 *μ*l PBS for enumeration of total white blood cells (WBC), lymphocytes (LYM), monocytes (MONO), neutrophils (NEU), and eosinophils (EOS) using a hemocytometer.

### 2.6. Histologic Assessment and Immunohistochemical Analysis of Lung Tissues

Mouse lung tissues were fixed, paraffin-embedded, cut, and stained with hematoxylin-eosin (H&E) for analysis of inflammatory cell infiltration as described previously [17]. In another experiment, lung sections were dewaxed and rehydrated. Antigen retrieval was performed by heated citrate. Sections were then incubated with anti-Syk and anti-p-Syk antibodies at 4°C overnight and incubated with HRP-conjugated secondary antibody. After staining with an ABC kit, positive staining was observed under a microscope and percentage of positively stained cells was evaluated, and the mean integrated optical density (MOD) was quantified by the Image-Pro Plus 7.0 software.

### 2.7. Human PBMC Isolation, Culture, Stimulation, and Treatment

Whole blood samples of healthy volunteers were obtained from the Clinical Laboratory Center, Beijing Friendship Hospital. Human PBMCs were isolated by gradient centrifugation over Lymphocyte Separation Medium and cultured in RPMI-1640 complete medium. The cells were then aliquoted into a 96-well culture plate at 2.5 × 10^5^/well and pretreated with different concentrations of PLE (25 *μ*g/ml, 50 *μ*g/ml, and 100 *μ*g/ml) and Dex (1 *μ*M). The cells were then stimulated twice with OVA [20] (0.5 *μ*g/ml) at 1 h and 12 h, respectively. The supernatants were then collected at 24 h to detect the levels of IL-6 and IL-8 production.

### 2.8. Approval for Human Experiments

The study involved with human sample was conducted in line with the guidelines outlined in the Declaration of Helsinki and had full ethical approval from the Institutional Ethics Committee of the Clinical Laboratory, Beijing Friendship Hospital. Informed consent was received from the volunteers.

### 2.9. RBL-2H3 Cell Culture, Stimulation, and Treatment

RBL-2H3 cells were cultured in DMEM medium supplemented with 10% FBS, 100 U/ml penicillin, and 100 *μ*g/ml streptomycin. The plated cells were sensitized with anti-DNP IgE (50 ng/ml) [21] for 12 h before being pretreated with different concentrations of treatments for 1 h. Then, the cells were induced with DNP-BSA (25 ng/ml) in PIPES buffer for 45 min, cells were used for toluidine blue staining, and cell culture supernatants were collected for *β*-Hex detection. Or the cells were induced with DNP-BSA (25 ng/ml) in complete DMEM medium for 4 h (cells were used for immunofluorescence (IF) staining and western blotting (WB) analysis) or 12 h (supernatants were collected for TNF-*α* detection).

### 2.10. *β*-Hexosaminidase Secretion Assay

After DNP-BSA stimulation, 0.05 ml cell supernatant was added to equal volume of p-nitrophenyl-N-acetyl-*β*-D-glucosaminide solution (2 mM, pH = 4.5) and incubated for 1 h at 37°C. Then, 0.2 ml stop solution (100 *μ*M NaHCO_3_/Na_2_CO_3_, pH = 10.0) was added to stop the reaction. The optical density value was read immediately at 405 nm. The release of *β*-Hex (%) was calculated as *β*‐Hex release (%) = the level of *β*‐Hex in each test well/the average level of *β*‐Hex in the model group × 100%.

### 2.11. Toluidine Blue Staining, IF Staining, and WB Analysis

Toluidine blue staining was used to visualize morphological changes of cells, and IF staining and WB were employed to determine the protein levels of Syk, p-Syk, PKC, p-PKC, NF-*κ*B p65, p-NF-*κ*B p65, cPLA_2_, and p-cPLA_2_. These experiments were performed as described previously [17].

### 2.12. ELISA Analysis

The concentrations of cytokines in BALF, culture supernatants from PBMCs and RBL-2H3 cells, and the levels of immunoglobulins in serum were detected by ELISA kits.

### 2.13. Statistical Analysis

Data were all expressed as mean ± standard error (SEM). As the normality test by Kolmogorov-Smirnov test (K-S test) was passed, data were analyzed by the Student *t*-test for comparison between two groups and one-way ANOVA for multiple groups followed by Fisher's least significant difference (LSD) test or otherwise by using the Kruskal-Wallis H test. *P* values less than 0.05 were considered as significant. The above analyses were conducted with GraphPad Prism (6.0) and IBM SPSS (19.0) statistical software.

## 3. Results

### 3.1. PLE Exerted Anti-Inflammatory Effect on Allergic Asthma *In Vivo*

**Figure 1:**
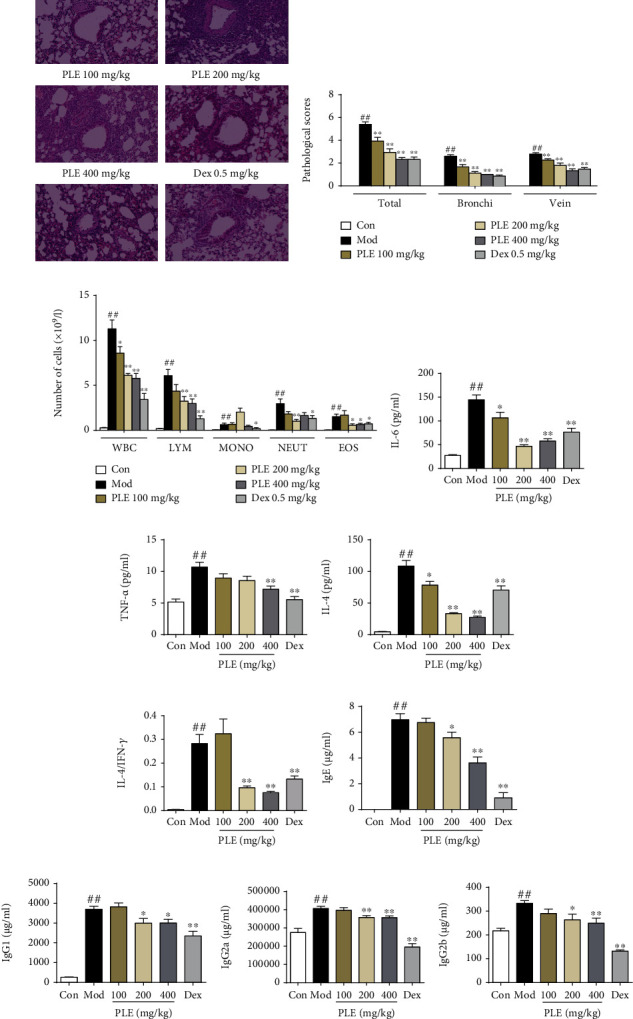
PLE exerted anti-inflammation effect in allergic asthma *in vivo*. (a) Representative images showing pathological changes in lung tissues determined by H&E staining (magnification, ×100). (b) The scores of inflammatory cell infiltration in lung tissues (*n* = 15 independent slices from three mice of each group). (c) WBC, LYM, MONO, NEUT, and EOS counts in BALF (*n* = 8). (d–g) The level of IL-6, TNF-*α*, and IL-4 and the ratio of IL-4/IFN-*γ* in BALF (*n* = 8). (h–k) The concentration of IgE, IgG1, IgG2a, and IgG2b in serum (*n* = 8). ^##^*P* < 0.01*vs.* the control group; ^∗^*P* < 0.05 and ^∗∗^*P* < 0.01*vs.* the model group.

PLE significantly attenuated the allergic airway inflammation in asthmatic mice induced by OVA. By using H&E staining (Figures 1(a) and 1(b)), the results of lung histologic changes showed that PLE treatment markedly attenuated OVA-induced extensive accumulation of inflammatory cells into bronchi and vein regions in a dose-dependent manner (*P* < 0.01). In agreement with the histologic appearance, PLE treatment significantly decreased the number of total leukocytes, lymphocytes, monocytes, neutrophils, and eosinophils in BALF of asthma mice (*P* < 0.05 or 0.01, Figure 1(c)). Furthermore, PLE treatment significantly reduced the production of IL-6 (Figure 1(d)), TNF-*α* (Figure 1(e)), and IL-4 (Figure 1(f)) in BALF as well as the level of IgE (Figure 1(h)), IgG1 (Figure 1(i)), IgG2a (Figure 1(j)), and IgG2b (Figure 1(k)) in serum (*P* < 0.05 or 0.01). Importantly, PLE treatment significantly decreased the level of IL-4, but had no effect on the level of IFN-*γ* (data not shown), resulting in a significant restoration of the IL-4/IFN-*γ* ratio imbalance (Figure 1(g)). This suggests that PLE can inhibit Th2 responses.

### 3.2. PLE Exerted Anti-Inflammatory Effect on Allergic Asthma *In Vitro*

**Figure 2:**
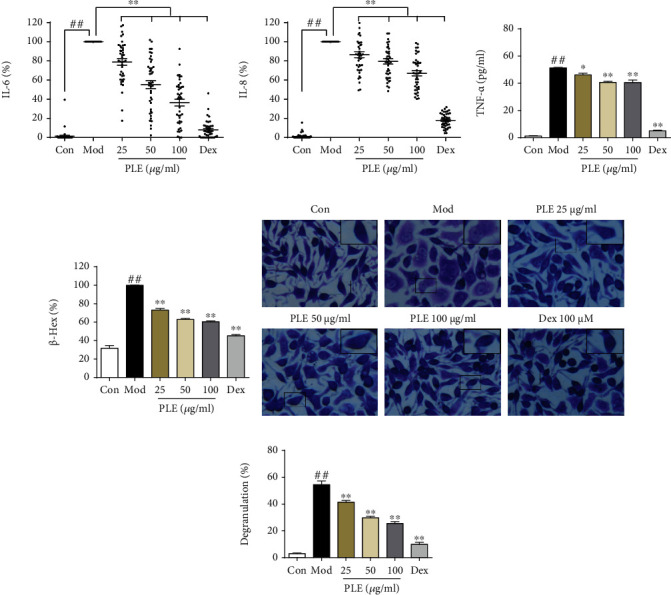
PLE exerted anti-inflammation effect in allergic asthma *in vitro*. (a, b) The levels of IL-6 and IL-8 production (%) in OVA-induced human PBMCs (*n* = 42). (c) The concentrations of TNF-*α* in DNP-IgE/BSA-induced RBL-2H3 cells (*n* = 3 independent experiments) measured by ELISA. (d) The level of *β*-Hex (%) in DNP-IgE/BSA-induced RBL-2H3 cells (*n* = 3 independent experiments). (e) Images of RBL-2H3 cells stained by toluidine blue (magnification, ×400) with (f) its degranulation rate (%) (*n* = 3). ^##^*P* < 0.01*vs.* the control group; ^∗^*P* < 0.05 and ^∗∗^*P* < 0.01*vs.* the model group.

PLE treatment also significantly suppressed the inflammatory responses in OVA-induced human PBMC allergic model and DNP-IgE/BSA-induced RBL-2H3 allergic cell model. The production of IL-6 (Figure 2(a)) and IL-8 (Figure 2(b)) was significantly increased in OVA-induced human PBMCs, which was significantly inhibited by PLE treatment (*P* < 0.01). In DNP-IgE/BSA-induced RBL-2H3 cells, PLE treatment significantly suppressed the production of TNF-*α* (*P* < 0.05 or 0.01, Figure 2(c)) and the release of *β*-Hex (*P* < 0.01, Figure 2(d)). In addition, by using toluidine blue staining, DNP-IgE/BSA induced the cellular morphological changes (DNP-IgE/BSA-induced cells became round or irregular shape, and some cells were vacuolated with diminished cytoplasm, in contrast to cells in the control group with spindle-shaped and closely packed secretory granules in the cytoplasm) and degranulation in RBL-2H3 cells, which were significantly inhibited with PLE treatment (*P* < 0.01, Figures 2(e) and 2(f)).

### 3.3. PLE Attenuated Allergic Airway Inflammation *In Vivo* by Inhibiting Syk and Its Downstream Mediators

**Figure 3:**
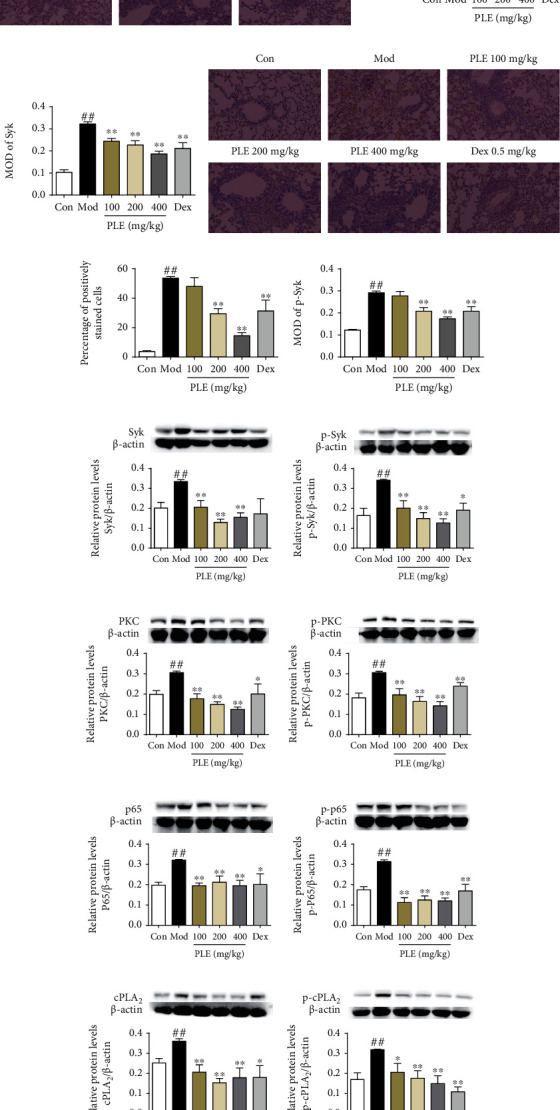
Effects of PLE on the expressions and phosphorylation of Syk and its downstream signaling molecules *in vivo*. (a) The representative photomicrographs (×200), (b) percentage of positively stained cells, and (c) mean integrated optical density (MOD) of Syk staining (*n* = 15 independent slices from three mice in each group). (d) Representative photomicrographs (×200), (e) percentage of positively stained cells, and (f) MOD of p-Syk staining (*n* = 15 independent slices from three mice in each group). The expression of (g) Syk, (h) p-Syk, (i) PKC, (j) p-PKC, (k) p65, (l) p-p65, (m) cPLA_2_, and (n) p-cPLA_2_ in OVA-induced asthma mouse lung tissues was determined by WB (*n* = 5). ^##^*P* < 0.01*vs.* the control group; ^∗^*P* < 0.05 and ^∗∗^*P* < 0.01*vs.* the model group.

The activation of Syk and its downstream mediators in lung tissues of asthma mice was significantly inhibited with PLE treatment. The positively stained cells and MOD of Syk and p-Syk in lung tissues were significantly decreased with PLE treatment (*P* < 0.01, Figures 3(a)–3(f)) as determined by immunohistochemical analysis, which were consistent with WB, showing significant inhibition in the expression of Syk and p-Syk (Figures 3(g) and 3(h)). Furthermore, PLE treatment inhibited the expression of PKC (Figure 3(i)), p-PKC (Figure 3(j)), p65 (Figure 3(k)), p-p65 (Figure 3(l)), cPLA_2_ (Figure 3(m)), and p-cPLA_2_ (Figure 3(n)) (*P* < 0.05 or 0.01) significantly in OVA-induced asthma mouse lung tissues.

### 3.4. PLE Attenuated Allergic Airway Inflammation *In Vitro* via Inhibiting Syk and Its Downstream Mediators

**Figure 4:**
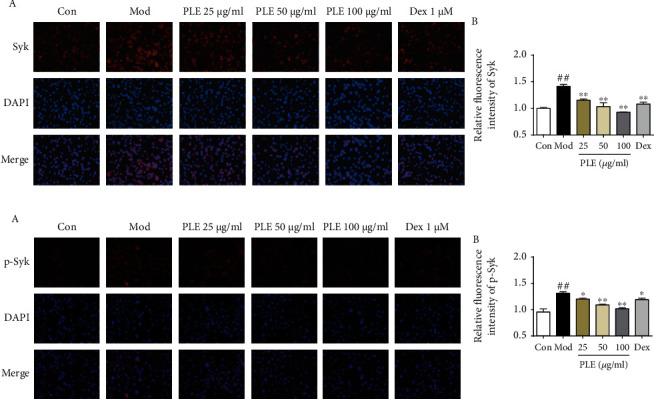
Effects of PLE on the expressions and phosphorylation of Syk in RBL-2H3 cells in vitro. (a) (A) The representative photomicrographs (magnification, ×200) of Syk and (B) their relative fluorescence intensity investigated by IF staining (*n* = 3). (b) (A) The representative photomicrographs (magnification, ×200) of p-Syk and (B) their relative fluorescence intensity investigated by IF staining (*n* = 3). ^##^*P* < 0.01*vs.* control group; ^∗^*P* < 0.05 and ^∗∗^*P* < 0.01*vs.* model group.

PLE treatment also significantly inhibited the expression and activation of Syk in DNP-IgE/BSA-induced RBL-2H3 cells. The expression of Syk, as determined by IF labeling, was significantly inhibited by PLE treatment in a dose-dependent manner (*P* < 0.01, Figure 4(a)). A similar result was observed for p-Syk antibody labeling (Figure 4(b)).

### 3.5. PLE Attenuated Allergic Airway Inflammation Associated with Syk and Its Downstream Signals PKC, NF-*κ*B, and cPLA_2_

**Figure 5:**
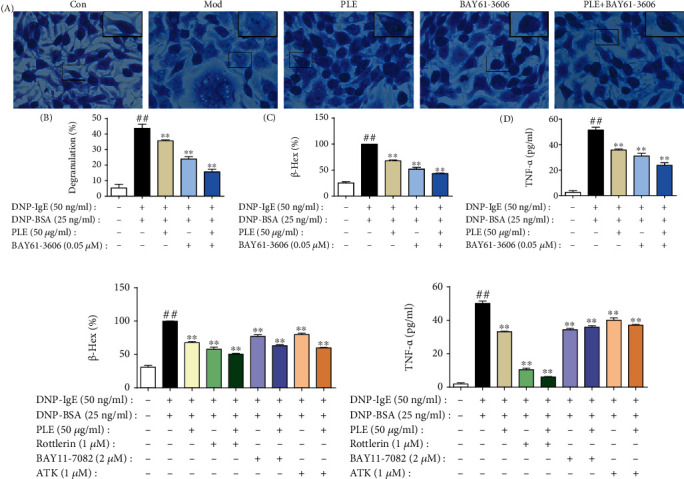
Verification that Syk, PKC, NF-*κ*B, and cPLA_2_ were related with PLE attenuated inflammation in DNP-IgE/BSA-induced RBL-2H3 cells. (a) (A) Images of RBL-2H3 cells stained by toluidine blue (magnification, ×400), (B) the percent of degranulation (%) (*n* = 3), (C) the release rate of *β*-Hex (%), and (D) the concentration of TNF-*α* in cells cotreated with PLE and Syk inhibitor (*n* = 3 independent experiments). (b) The release rate of *β*-Hex and (c) production of TNF-*α* in cells cotreated with PLE and PKC, NF-*κ*B, or cPLA_2_ inhibitor (*n* = 3 independent experiments). ^##^*P* < 0.01*vs.* the control group; ^∗^*P* < 0.05 and ^∗∗^*P* < 0.01*vs.* the model group.

To examine the role of Syk by which PLE attenuates allergic airway inflammation, inhibitors of Syk (BAY61-3606) and its downstream signals PKC (Rottlerin), NF-*κ*B (BAY11-7082), and cPLA_2_ (ATK) were employed. Treatment with BAY61-3606 significantly inhibited the degranulation and TNF-*α* release in DNP-IgE/BSA-induced RBL-2H3 cells, which were collaboratively inhibited by cotreating with PLE (Figure 5(a)). In addition, PLE cotreatment with Rottlerin, BAY11-7082, and ATK collaboratively inhibited *β*-Hex (Figure 5(b)) along with TNF-*α* (Figure 5(c)) production in DNP-IgE/BSA-induced RBL-2H3 cells, respectively.

### 3.6. PLE Attenuated Allergic Airway Inflammation by Targeting Syk

**Figure 6:**
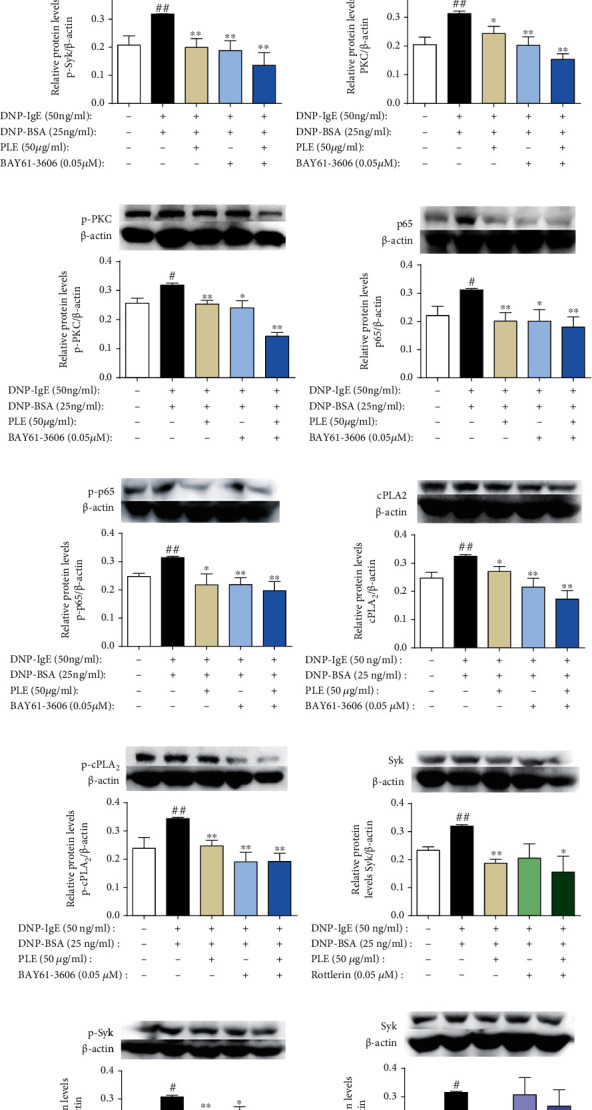
PLE attenuated allergic inflammation by targeting Syk in DNP-IgE/BSA-induced RBL-2H3 cell *in vitro*. (a) (A) Images (magnification, ×200) of Syk expression and (B) their relative fluorescence intensities determined by IF staining (*n* = 3). (b) The expression of Syk cotreating with PLE and BAY61-3606 detected by WB (*n* = 5). (c) (A) Images of (magnification, ×200) p-Syk expression and (B) their relative fluorescence intensities determined by IF staining (*n* = 3). (d) The expression of p-Syk cotreating with PLE and BAY61-3606 detected by WB (*n* = 5). (e–j) The expression of PKC, p-PKC, p65, p-p65, cPLA_2_, and p-cPLA_2_ cotreating with PLE and BAY61-3606 detected by WB (*n* = 5). (k–p) The expression of Syk and p-Syk cotreating with PLE and Rottlerin, BAY11-7082, and ATK detected by WB (*n* = 5). ^##^*P* < 0.01*vs.* the control group; ^∗^*P* < 0.05 and ^∗∗^*P* < 0.01*vs.* the model group.

PLE cotreatment with BAY61-3606 almost collaboratively abolished the expression of Syk (Figure 6(a)) and p-Syk (Figure 6(c)) determined by IF staining, which were further confirmed with WB (Figures 6(b) and 6(d)). Interestingly, PLE decreased the expression of PKC, NF-*κ*B, and cPLA_2_, which were superimposed by cotreating with BAY61-3606 (Figures 6(e)–6(j)). However, except Rottlerin, BAY11-7082 and ATK did not inhibit the protein expression of Syk and p-Syk (Figures 6(k)–6(p)). The data collectively revealed that Syk was the important target through which PLE mediated allergic inflammatory response.

## 4. Discussion

Allergic asthma is a chronic inflammatory lung disease with a steadily increasing incidence and poses a health challenge to people worldwide. Owing to the drug resistance and adverse reactions of current available anti-inflammation treatment, new managements are still needed for airway inflammation of allergic asthma. The results of the present study demonstrate that PLE, an extract from Perilla leaves, has a significant antiallergic airway inflammation property *in vivo* and *in vitro.*

Importantly, data in this study suggested PLE might notably reduce inflammatory mediators and alleviate immediate allergic response. PLE regulated OVA-induced mouse allergic inflammation responses, including inhibiting infiltration of inflammatory cells in lung tissues, reducing the accumulation of inflammatory cells (lymphocytes, macrophages, neutrophils, and eosinophils) in the BALF, and decreasing the production of inflammatory cytokines (IL-6, TNF-*α*, and IL-4) in the BALF and the secretion immunoglobulin (IgE, IgG1, IgG2a, and IgG2b) in serum. Meanwhile, PLE treatment decreased the inflammatory cytokine (IL-6 and IL-8) production in OVA-induced human PBMCs and attenuated degranulation and TNF-*α* secretion in antigen-induced RBL-2H3 cells. And PLE treatment also significantly decreased the level of IL-4 and the ratio of IL-4/IFN-*γ*, suggesting a regulatory effect on Th1/Th2 imbalance which causes the pathogenesis of asthma [22].

Further, we investigated potential target and mechanism by which PLE regulated the allergic airway inflammation. Asthma occurs as type I hypersensitivity reactions when antigens bind to the IgE-Fc*ε*RI complex and then recruit and activate Syk [23], which is known to involve in regulation of allergic inflammation response [24] and has been recognized as a potential target for the treatment of immune-mediated disorders such as asthma [25]. In respiratory allergies, upregulated Syk results in the activation of downstream signaling molecules, including PKC, NF-*κ*B, and cPLA_2_, leading to the release of cytokines and inflammatory mediators [16, 26]. Thus, Syk can be an attractive target for therapeutic intervention in asthma. The results of our study showed that PLE treatment significantly suppressed the expression of Syk and p-Syk both *in vivo* and *in vitro*. This indicated that PLE exerted anti-inflammatory effects possibly by inhibiting the phosphorylation and expression of Syk.

Also, PKC, NF-*κ*B, and cPLA_2_ levels were detected *in vivo* and *in vitro*. PKC is a well-known family of homologous serine/threonine kinases associated with asthma pathogenesis including airway inflammation, tissue injury, and remodeling [27–29]. NF-*κ*B has a viral role in the inflammatory networks of asthma by regulating the expression of cytokines, chemokines, adhesion molecules, and infiltrating inflammatory cells [30, 31]. cPLA_2_ is responsible for the process of asthma through producing arachidonic acid, which is subsequently metabolized into inflammatory mediators such as prostaglandins, thromboxanes, and leukotrienes, resulting in airway eosinophilia and bronchoconstriction [32]. Our data showed that PLE suppressed the expression and phosphorylation of PKC, NF-*κ*B, and cPLA_2_*in vivo* and *in vitro*, suggesting that PLE may exert its protective effects partly through mediating PKC, NF-*κ*B, and cPLA_2_.

Furthermore, by cotreating PLE with BAY61-3606 (Syk inhibitor), Rottlerin (PKC inhibitor), BAY11-7082 (NF-*κ*B inhibitor), and ATK (cPLA_2_ inhibitor) in DNP-IgE/BSA-stimulated RBL-2H3 cells, respectively, it was demonstrated that Syk might be a potential target through which PLE could alleviate the inflammatory response via modulating its downstream signaling molecules PKC, p65, and cPLA_2_. By cotreating PLE with BAY61-3606, a further extent of inhibition in the expression and phosphorylation of Syk, PKC, NF-*κ*B, and cPLA_2_ was observed, while BAY11-7082 and ATK did not impact the expression of Syk and p-Syk. Cotreating Rottlerin with PLE suggested a mutual interaction between Syk and PKC with PLE treatment.

## 5. Conclusion

In conclusion, PLE can significantly prevent allergic airway inflammation *in vivo* and *in vitro* by targeting Syk and then regulating its downstream signals PKC, p65, and cPLA_2_.

## Data Availability

The data that support the findings of this study are available from the corresponding author upon reasonable request.

## Abbreviations

PLE: Perilla leaf extract
ATK: Arachidonyl trifluoromethyl ketone.
